# Behavioral Influence of Known Prognostic Markers on the Cardiologist's Decision following Acute Coronary Syndrome: the GRACE Score Paradox

**DOI:** 10.5935/abc.20190046

**Published:** 2019-06

**Authors:** Manuela Campelo Carvalhal, Thiago Menezes Barbosa de Souza, Jessica Suerdieck, Fernanda Lopes, Vitor Calixto de Almeida Correia, Yasmin Falcon Lacerda, Nicole de Sá, Gabriella Sant'Anna Sodré, Marcia Maria Noya Rabelo, Luis Cláudio Lemos Correia

**Affiliations:** 1 Escola Bahiana de Medicina e Saúde Pública, Salvador, BA - Brazil; 2 Hospital São Rafael, Fundação Monte Tabor, Salvador, BA - Brazil

**Keywords:** Acute Coronary Syndrome, Prognosis, Non-ST Elevation Myocardial Infarction

## Abstract

**Background:**

Behavioral scientists consistently point out that knowledge does not influence decisions as expected. GRACE Score is a well validated risk model for predicting death of patients with acute coronary syndromes (ACS). However, whether prognostic assessment by this Score modulates medical decision is not known.

**Objective:**

To test the hypothesis that the use of a validated risk score rationalizes the choice of invasive strategies for higher risk patients with non-ST-elevation ACS.

**Methods:**

ACS patients were consecutively included in this prospective registry. GRACE Score was routinely used by cardiologists as the prognostic risk model. An invasive strategy was defined as an immediate decision of the coronary angiography, which in the selective strategy was only indicated in case of positive non-invasive test or unstable course. Firstly, we evaluated the association between GRACE and invasiviness; secondly, in order to find out the actual determinants of the invasive strategy, we built a propensity model for invasive decision. For this analysis, a p-value < 0.05 was considered as significant.

**Results:**

In a sample of 570 patients, an invasive strategy was adopted for 394 (69%). GRACE Score was 118 ± 38 for the invasive group, similar to 116 ± 38 for the selective group (p = 0.64). A propensity score for the invasive strategy was derived from logistic regression: positive troponin and ST-deviation (positive associations) and hemoglobin (negative association). This score predicted an invasive strategy with c-statistics of 0.68 (95%CI: 0.63-0.73), opposed to GRACE Score (AUC 0.51; 95%CI: 0.47-0.57).

**Conclusion:**

The dissociation between GRACE Score and invasive decision in ACS suggests that the knowledge of prognostic probabilities might not determine medical decision.

## Introduction

The risk-treatment paradox is a common phenomenon in which, contrary to what is expected, patients with higher risk receive less aggressive treatment as compared with individuals with lower risk.^1^ One of the causes of this paradox is an equivocal risk evaluation based on the physician's intuitive impression. Probabilistic risk models have shown to be more accurate than intuitive judgment, suggesting that the use of such models theoretically facilitates prognosis-based treatment choice.^2-4^

However, behavioral scientists have demonstrated that knowledge does not modulate decisions as expected.^5^ In economy, people tend to make irrational decisions, which is not different in health-related issues. For example, it is well known smoking or obesity are risk factors for serious diseases, but habits of smoking, or eating improperly are common. Therefore, whether the use of a risk score actually modulates the physician's decision is unknown.

Non-ST-segment elevation acute coronary syndromes (ACS) present with a wide spectrum of risks, and patients can be treated in a conservative or aggressive manner.^6,7^ This is one of the main clinical scenarios in which the risk-treatment paradox has been described.^8^ Even though GRACE Score is a well-validated risk model for patients with ACS, its actual impact on providing a more reasonable approach according to risk, and on its relationship with medical judgment, has yet to be demonstrated.^9,10^ Our aim was to test the hypothesis that the utilization of a risk score rationalizes the choice for invasive strategies towards higher risk patients with with non-ST elevation acute coronary syndromes.

## Methods

### Sample selection

Patients consecutively admitted to the coronary care unit (CCU) of a tertiary-care hospital due to non-ST elevation acute coronary syndromes between August 2007 and October 2014 were included in the study. Inclusion criteria was typical chest discomfort plus at least 1 of the 3 objective criteria: electrocardiographic changes consisting of transient ST-segment depression (0.05 mV), or T wave inversion (0.1 mV); troponin change to a level beyond the 99^th^ percentile threshold of a healthy reference population, with 10% coefficient of variability;^11^ or previous documentation of coronary artery disease, defined as a definitive history of myocardial infarction, or coronary obstruction ≥ 50% at angiography. Patient's option not to participate in the Registry was the sole exclusion criteria. All participants provided written informed consent.

### Study protocol

Patients included were classified as for invasive or selective strategies according to medical decision. Management strategy was decided by the cardiology team in the CCU and was not influenced by the study protocol. Invasive strategy was prospectively defined by a decision to perform invasive coronary angiography, followed by a revascularization procedure if anatomically indicated. Selective strategy was defined as an indication of angiography conditioned to a positive non-invasive test, or clinical instability.

GRACE Score was used for evaluation of baseline risk, defined by tertiles of the original study (low risk: 1-108; intermediate risk: 109-140; high risk: 141-372). Death during hospitalization was the outcome of interest.

### Statistical analysis

In order to evaluate whether baseline risk influenced the physician's decision regarding management strategy, GRACE Score was compared between the groups undergoing invasive versus selective strategy by the Mann-Whitney statistic. Secondly, in order to understand the determinants of medical decision, logistic regression was utilized to assess independent predictors of the invasive strategy. The selection of variables for this analysis was based on their univariate association with the invasive strategy (p < 0.10). A propensity score for the invasive strategy was derived from the logistic regression. Thirdly, in order to evaluate whether medical decision was correctly driven by prognosis, the value of the propensity score for predicting death during hospitalization was tested by the C-statistics (area under the ROC curve). C-statistics of the propensity score was compared with the c-statistics of GRACE Score by Hanley-Mcneil's test.

The analysis of normality was done through the combination of histogram and Q-Q plots visualization, description of skewness and kurtosis with confidence intervals and normality tests (Shapiro-Wilk and Kolmogorov-Smirnov). Numeric variables were expressed by means (standard deviation) or medians (interquartile range), and compared by unpaired student's t test or Mann-Whitney test. Categorical variables were described by frequencies and compared by Pearson's chi-square test, or Fisher's exact test. SPSS Statistical Software (Version 21, SPSS Inc., Chicago, Illinois, USA) was utilized for data analysis.

## Results

A sample of 570 consecutive patients admitted with non-ST-segment elevation ACS was studied, aged 69 ± 14 years, 50% males. GRACE Score had a normal distribution, with mean of 118 ± 38. According to GRACE definition, 46% of patients were defined as low risk, 30% as intermediate risk, and 24% as high risk. Management through an invasive strategy took place in 69% of the patients.

**Figure 1:**
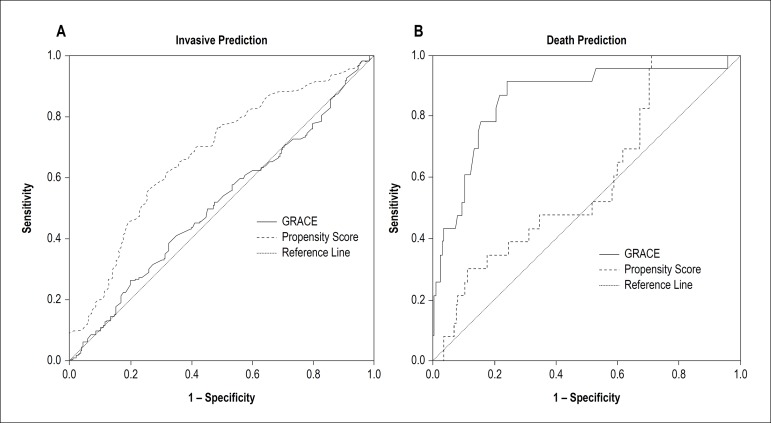
According to the area under the curve, GRACE Score does not predict an invasive strategy, as opposed to the propensity score (Panel A, p < 0.001 for curve comparison). Conversely, GRACE Score is better than the propensity Score for the prediction of mortality (Panel B, p < 0.001).

GRACE Score of patients who underwent an invasive strategy was 118 ± 38, similar to patients managed conservatively (116 ± 38; p = 0.64). Seemingly, the area under the ROC curve for GRACE Score predicting an invasive strategy was not significant (0.51; 95% CI = 0.47 - 0.57; p = 0.51) - Figure 1A. There was no difference in the frequency of invasive strategy among patients with low, intermediate and high risk according to GRACE (68%, 77%, 73%, respectively; p = 0.48).

Table 1 depicts univariate association between patients' characteristics and management strategies. Among GRACE variables, Killip class, systolic blood pressure, heart rate, and creatinine did not have any association with the strategy chosen. On the contrary, positive troponin (OR = 2.7; 95% CI = 1.8 - 3.8; p < 0.001), ST-deviation (OR = 2.0; 95% CI = 1.2 - 3.2; p = 0.006), and the numeric value of hemoglobin at admission (OR = 1.2; 95% CI = 1.1 - 1.4; p < 0.001) predicted an invasive strategy. Conversely, age as a numeric variable had an inverse relationship with invasive strategy (OR = 0.98; 95% CI = 0.97 - 0.99; p < 0.013). Finally, the risk of bleeding according to CRUSADE Score was protective against invasive strategy (OR = 0.98; 95% CI = 0.97 - 0.99; p < 0.018).

**Table 1:** Exploratory analysis of variable associates with strategy

	Chosen Strategy		p value
	Invasive	Selective	
Sample Size	394	176	
Male Gender	204 (52%)	82 (47%)	0.25*
Age (years)	66 ± 14	69 ± 14	0.01†
Positive Troponin	249 (63%)	69 (39%)	< 0.001*
ST Depression	94 (24%)	24 (14%)	0.005*
Killip > 1	57 (15%)	24 (14%)	0.81*
LV Ejection Fraction < 45%	26 (7.3%)	12 (7.8%)	0.84*
Systolic BP (mmHg)	154 ± 28	155 ± 33	0.68†
Heart Rate (bpm)	79 ± 20	77 ± 16	0.30†
Creatinine (mg/dl)	1.1 ± 0.84	1.2 ± 1.1	0.35†
Diabetes	143 (36%)	62 (35%)	0.79*
Smoking	33 (8.4%)	11 (6.3%)	0.38*
Number of Risk Factors	2.2 ± 1.0	2.1 ± 1.1	0.21†
Known Coronary Artery Disease	209 (53%)	104 (59%)	0.19*
Hemoglobin	13.4 ± 1.8	12.7 ± 2.1	< 0.001†
CRUSADE Bleeding Score	38 ± 15	41 ± 14	0.02†

Known Coronary Artery Disease =Definitive history of myocardial infarction or coronary obstruction ≥50% at angiography; LV: left ventricle. BP: blood pressure.

* Pearson's chi-square test p-values;

† Unpaired Student's T test p-values.

A logistic regression model was used to build a propensity score for invasive strategy. The 5 variables associated with the invasive strategy in a univariate analysis were included. Positive troponin (OR = 2.5; 95% CI = 1.7 - 3.7; p < 0.001), ST-deviation (OR = 1.8; 95% CI = 1.1 - 3.1; p = 0.026), and hemoglobin on admission remained positively associated (OR = 1.2; 95% CI = 1.1 - 1.4; p < 0.001). Age and CRUSADE Score lost statistical significance (p = 0.09 and 0.29, respectively) - Table 2. This propensity model was statistically significant (chi-square = 48; p < 0.001; R^2^ = 0.2), calibrated (H-L c^2^ = 12; p = 0.17), and had an area under the ROC curve (AUC) of 0.68 (95% CI = 0.63 - 0.73; p < 0.001) for predicting an invasive strategy. This AUC was significantly better than GRACE Score area for the strategy prediction (p < 0.001) - Figure 1A.

**Table 2:** Logistic regression univariate and multivariate associations between the candidate's predictive variables and invasive strategy

	Univariate Analysis		Multivariate Analysis			
			Model 1		Model 2	
	OR (95% CI)	p Value	OR (95% CI)	p Value	OR (95% CI)	p Value
Positive Tn	2.7 (1.8 - 3.8)	< 0.001	2.5 (1.7 - 3.7)	< 0.001	2.6 (1.8 - 3.8)	< 0.001
ST-deviation	2.0 (1.2 - 3.2)	0.006	1.8 (1.1 - 3.1)	0.026	1.8 (1.1 - 2.9)	0.026
Hemoglobin	1.2 (1.1 - 1.4)	0.001	1.2 (1.1 - 1.4)	< 0.001		--
Age	0.98 (0.97-0.99)	0.013	--	0.09	0.98 (0.96 - 0.99)	0.002
CRUSADE	0.98 (0.97-0.99)	0.018	--	0.29		--

The 5 variables on this table are the ones that reached statistical significance in univariate analysis. Model was derived by the initial inclusion of all 5 variables (full model) and Model 2 only included typical risk prediction variables (did not include hemoglobin and CRUSADE Score). Positive Tn = Troponin change to a level beyond the 99th percentile.

A secondary model was built only with variables commonly utilized as part of a risk profile in ACS patients. In this model, hemoglobin and CRUSADE were not included, making age an inversely associated independent predictor of invasive strategy, and positive troponin and ST-deviation positively associated with invasive strategy - Table 2.

The incidence of death during hospitalization was 5.1% (29 individuals). GRACE Score accurately predicted mortality, with an AUC of 0.87 (95% CI = 0.80 - 0.94; p < 0.001). The propensity score for invasive strategy also predicted mortality (AUC = 0.64; 95% CI = 0.56 - 0.72), but had a lower accuracy in comparison with GRACE Score (p < 0.001) - Figure 1B.

## Discussion

The present study found a dissociation between the risk predicted by a probabilistic model and the physician's choice towards invasive strategy in patients with non-ST-elevation acute coronary syndromes. GRACE Score was the probabilistic model utilized in this analysis, a well-validated and accurate tool for prediction of death in ACS.^9,10^ The study took place in an environment whose team of physicians has the duty to calculate GRACE Score for risk stratification and decision making. In spite of that, GRACE Score was not higher in individuals who underwent an invasive strategy, in comparison with patients of a selective strategy. Our findings reproduce behavioral science experiments where decisions are not well driven by knowledge.^5^

Contrary to GRACE Score, some patients' characteristics were independently associated with decision and were utilized to build a propensity score for invasive strategy. This score had a prognostic value lower than GRACE Score. Therefore, we found a paradox in which the variables that determined an invasive approach had a weaker association with prognosis in comparison with a true prognostic model that was not related to this decision.

Our findings are in line with previous evidences of dissociation between risk and intensity of treatment, the so-called risk-treatment paradox.^12-14^ This phenomenon takes place when management has a risk/benefit trade-off, and the size of beneficial effect correlates with risk of unintended consequences. In this case, individuals who mostly need the treatment are the ones who most discourage the physician's decision .^15^ For example, older ages were associated with a more conservative strategy, despite being the most important risk predictors in GRACE Score.^16,17^

Traditionally, medical judgment is based on intuition and experience, the so-called *gestalt.* This non-structured method of decision is vulnerable to cognitive bias.^18,19^ Possibly, in elderly patients, a kind of nihilistic view makes the sense of risk surpass the sense of beneficial effect, while there is more enthusiasm towards young individuals, making the sense of benefit surpass the sense of risk. The utilization of a probabilistic model tends to avoid under- or overestimation of probabilities due to cognitive bias. Instead, it allows the quantification and balance of the risk/benefit ratio. Secondly, it is proved in different scenarios that the estimation of probabilities under uncertainty is more accurate when a probabilistic model is utilized instead of gestalt.^19^ Indeed, in acute coronary syndromes, GRACE Score has shown to have better accuracy than the physician's opinion.^20,21^ Our data validates this concept, since GRACE Score was more accurate in relation to the propensity score for invasiveness.

However, a mental reluctance of specialists to utilize a mathematical model, at the expense of unstructured judgment, has been reported.^22^ Our observation is peculiar because it arises from an environment in which GRACE Score is systematically calculated and registered in the chart. In spite of that, physicians did not seem to be influenced by the predictive model, a phenomenon illustrated by GRACE Score being virtually identical in invasive and non-invasive groups. One could find only natural that physicians sometimes overrule GRACE Score based on patients' individualities and preferences. However, this should not be frequent enough to totally blunt the contrast of risk between the selective and invasive groups.

In our observations, positive troponin and ST-deviation were independent predictors of invasive strategy. They are both part of the 8 variables in GRACE Score, which were not associated with decision. This may be an indication that medical decision tends to be more univariate than multivariate, more deterministic than probabilistic.^14^ Probably, either a positive troponin or an ST-deviation would lead them to opt for the invasive strategy, as opposed to a multivariable probabilistic approach. Also, in our first model, low hemoglobin was independently associated with a more conservative strategy. Considering that hemoglobin is not a traditional prognostic marker, it may be acting as a proxy to a more fragile patient or one with more co-morbidities.

The limitation of our study is the generalization from a single CCU. Actually, we utilized our Unit as a model to test the hypothesis that the use of GRACE Score influences decision towards a more aggressive approach. While our study should not be generalized as a demonstration that decision-making has not been properly based on risk, it is an evidence that the utilization of a risk model does not guarantee risk-based decision. Moreover, our observation is in line with previous evidences of risk-treatment paradox.^14,23^ Finally, our findings only generate hypotheses to be further validated by a clinical trial, in which individuals would be allocated to utilization or no utilization of GRACE Score, and the frequency of the invasive strategy would be compared between the groups.

## Conclusion

In conclusion, the dissociation between GRACE Score and invasive decision in ACS suggests that the utilization of a prognostic model does not guarantee a risk-based decision.
